# Supplementary material for: Identification of replication fork-associated proteins in Drosophila embryos and cultured cells using iPOND coupled to quantitative mass spectrometry
Source: Sci Rep. 2022 Apr 28;12:6903. doi: 10.1038/s41598-022-10821-9 (PMC9050644; doi:10.1038/s41598-022-10821-9)

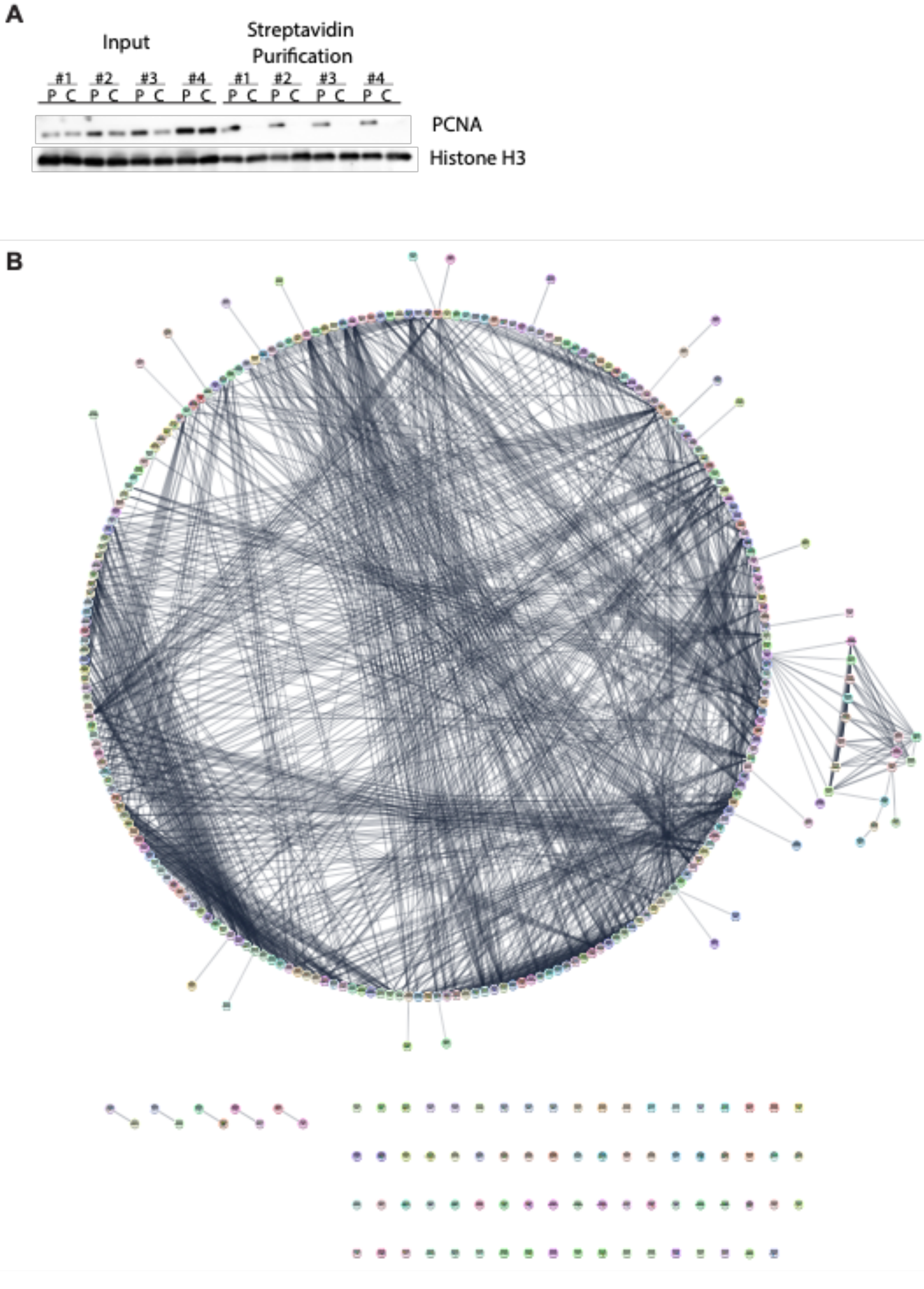

A
Input
Streptavidin Purification
#1 #2 #3 #4 #1 #2 #3 #4
P C P C P C P C P C P C P C P C
PCNA
Histone H3
B

Supplemental Figure 1. Analysis of iPOND and iPOND-TMT in S2 cultured cells. (**A**) Western blot confirmation of iPOND in S2 cells. The first eight lanes are from input and the last eight from the streptavidin purifications. PCNA is a marker for active replication forks and is enriched in the pulse sample purifications. Histone H3 is a general marker of chromatin and enriched in both the pulse and chase samples. P = Pulse, C = Chase, and #1-4 represent the replicate numbers. (**B**) Total network map of the 278 replisome-associated proteins in S2 cells.

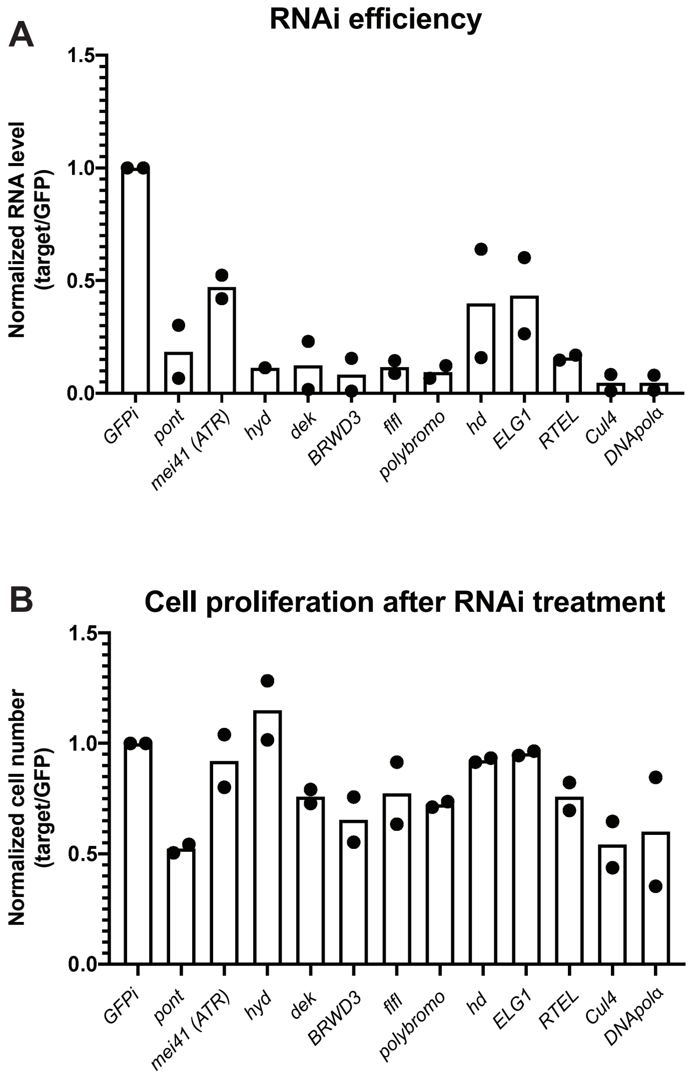

Supplemental Figure 2: Validation of RNAi-based depletion of targets. **(A)** Normalized depletion efficiency for two biological replicates. The normalized ratio is the target/*Tubulin* in the non-targeting *GFP* control divided by target/*Tubulin* in the RNAi-treated cells **(B)** Cell proliferation after five days of RNAi depletion relative to the *GFP* non-targeting control.

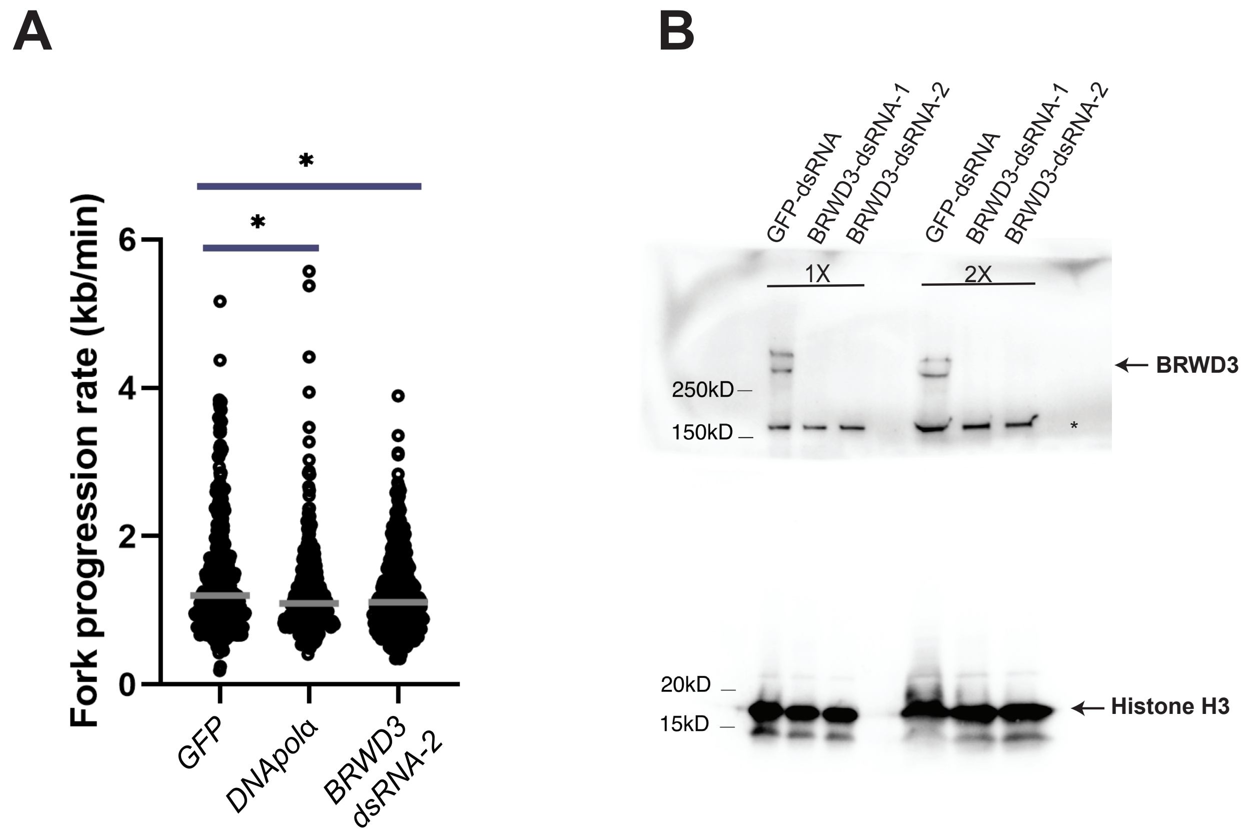

Supplemental Figure 3: Validation of *BRWD3* RNAi constructs. **(A)** Rate of fork progression in control and BRWD3 dsRNA-2 depleted S2 cells. 400 fibers from a single replicate were measured. Bars represent the median fork speed. * p<0.05 using a Kruskal-Wallis one-way analysis of variance followed by a Dunn's multiple comparison post-test. **(B)** Western validation of S2 cells treated two independent RNAi constructs targeting *BRWD3* or *GFP* as a negative control. Anti-BRWD3 (top) and anti-Histone H3 loading control (bottom). 1X and 2X indicates the relative amount of protein loaded. * marks a non-specific band in the anti-BRWD3 blot.

Uncropped gel image for anti-histone H3 Western blot in Figure 1D

Blot with MW markers

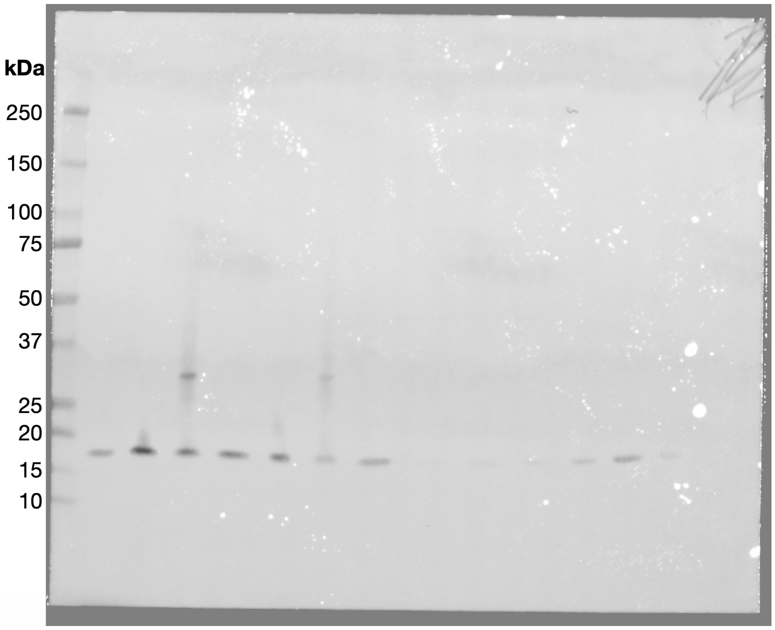

Blot alone

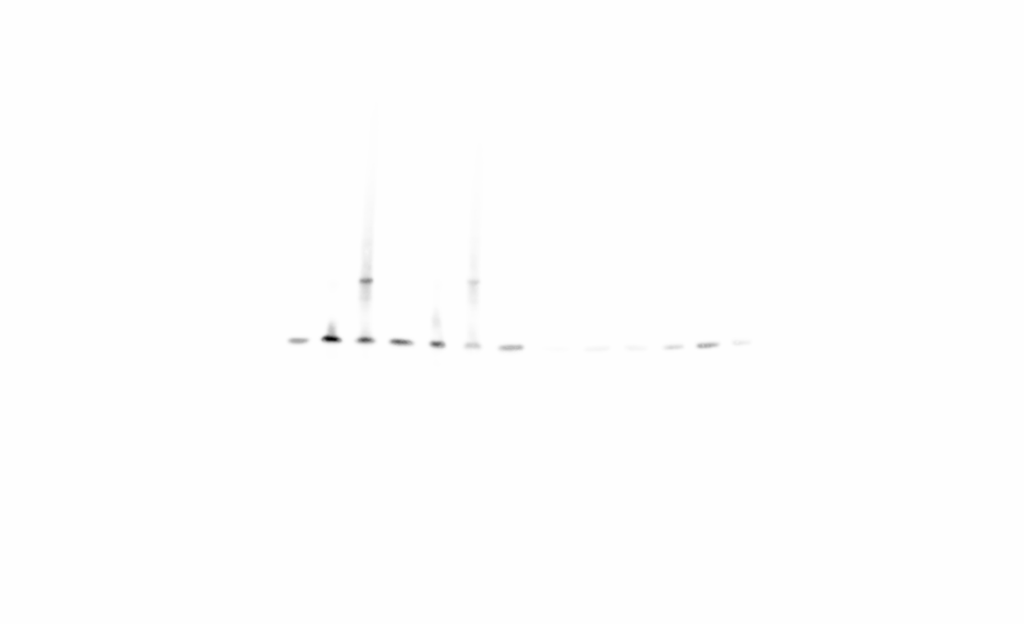

Uncropped gel image for anti-PCNA Western blot in Supplemental Figure 1A

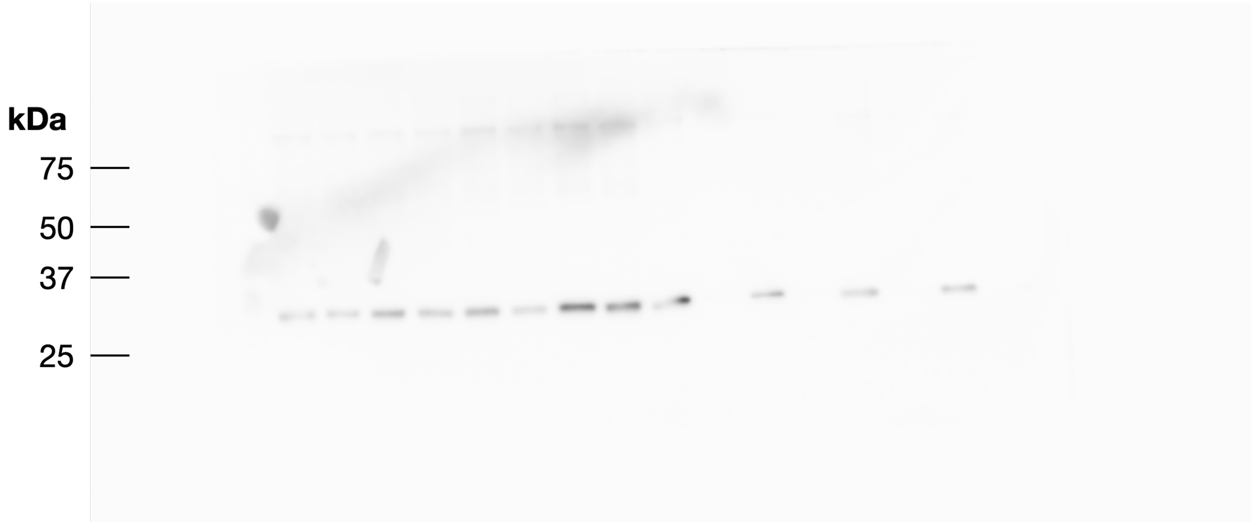

Uncropped gel image for anti-histone H3 Western blot in Supplemental Figure 1A

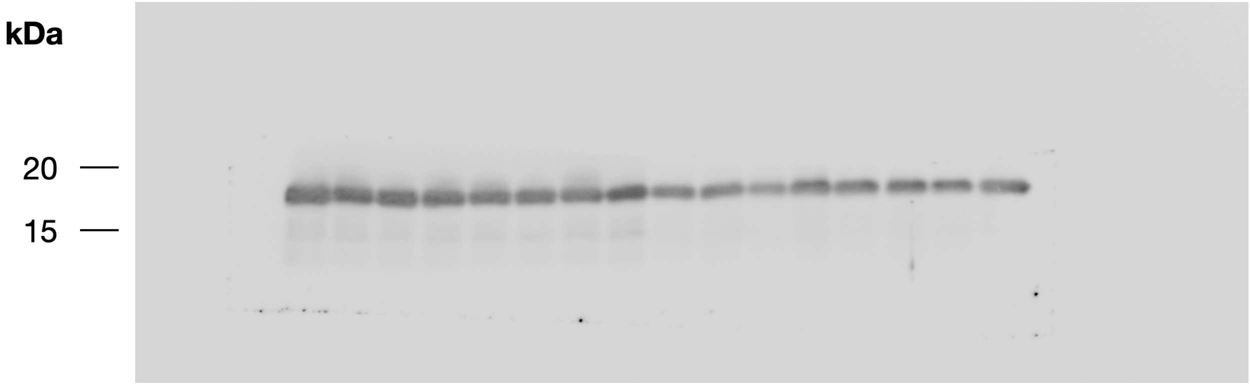

Uncropped gel image for anti-BRWD3 Western blot in Supplemental Figure 3B

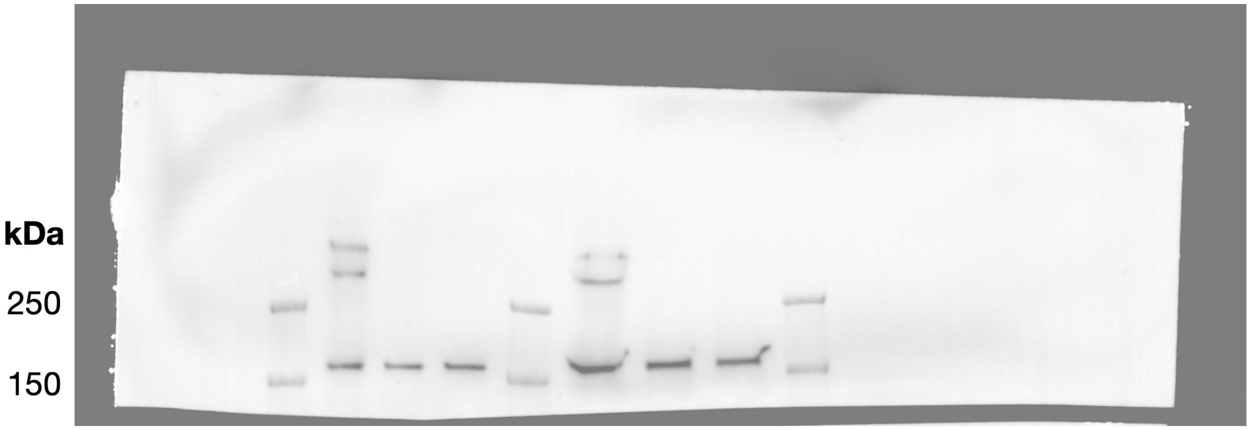

Uncropped gel image for anti-histone H3 Western blot in Supplemental Figure 3B

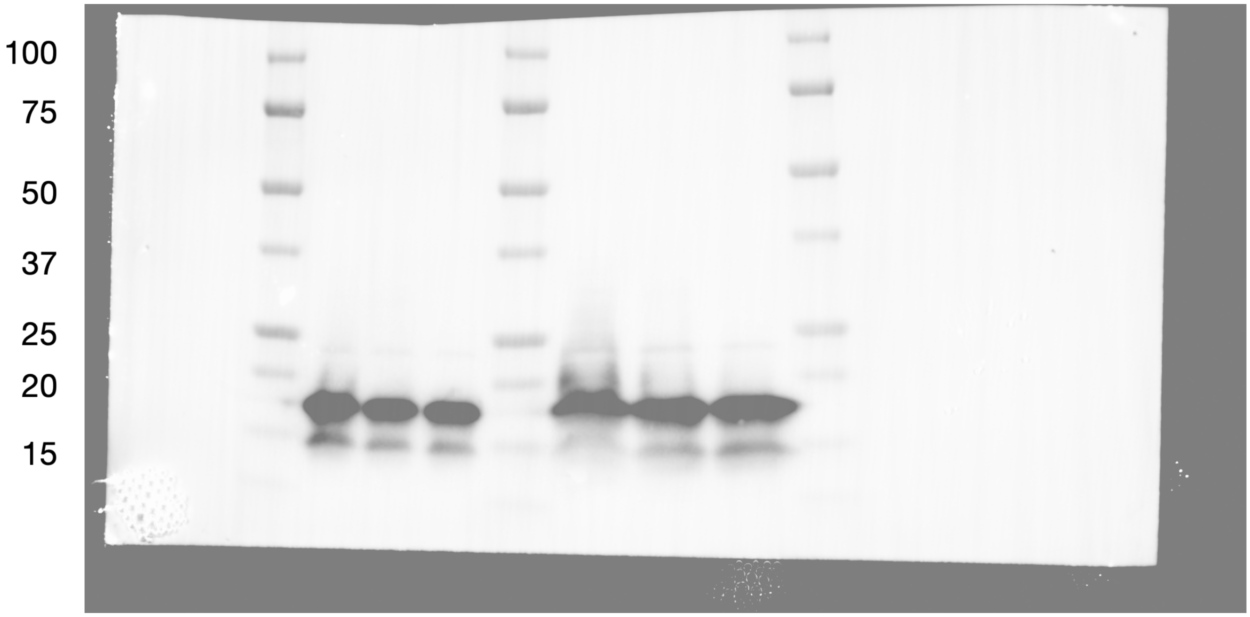

Supplement: Supplementary file 1 — Supplementary Figures. [file 41598_2022_10821_MOESM1_ESM.pdf]
